# Infrared-active phonons in one-dimensional materials and their spectroscopic signatures

**DOI:** 10.1038/s41524-023-01140-2

**Published:** 2023-10-18

**Authors:** Norma Rivano, Nicola Marzari, Thibault Sohier

**Affiliations:** 1 Theory and Simulations of Materials (THEOS), École Polytechnique Fédérale de Lausanne, CH-1015 Lausanne, Switzerland; 2 National Centre for Computational Design and Discovery of Novel Materials (MARVEL), École Polytechnique Fédérale de Lausanne, CH-1015 Lausanne, Switzerland; 3 Laboratory for Materials Simulations, Paul Scherrer Institut, 5232 Villigen PSI, Switzerland; 4 Laboratoire Charles Coulomb (L2C), Université de Montpellier, CNRS, Montpellier, France

**Keywords:** Nanoscale materials, Condensed-matter physics

## Abstract

Dimensionality provides a clear fingerprint on the dispersion of infrared-active, polar-optical phonons. For these phonons, the local dipoles parametrized by the Born effective charges drive the LO-TO splitting of bulk materials; this splitting actually breaks down in two-dimensional materials. Here, we develop the theory for one-dimensional (1D) systems—nanowires, nanotubes, and atomic and polymeric chains. Combining an analytical model with the implementation of density-functional perturbation theory in 1D boundary conditions, we show that the dielectric splitting in the dispersion relations collapses as ${x}^{2}\log (x)$ at the zone center. The dielectric properties and the radius of the 1D materials are linked by the present work to these red shifts, opening infrared and Raman characterization avenues.

## Introduction

Phonons and their interactions with electrons and photons are key ingredients in determining the thermodynamic, transport, and optical properties of materials^1,2^. Notably, long-wavelength optical phonons can give rise to electric fields which strongly affect not only their dispersion relations^3–8^, but also the physics of Fröhlich electron-phonon interactions^9–11^ and phonon polaritronics^12^. In semiconductors and insulators, when atoms in the lattice have non-vanishing Born effective charges (BECs), optical phonons can generate a polarization density and couple with electric fields. Those modes are then termed polar and are infrared active. In addition, for longitudinal atomic displacement patterns, and in particular for purely longitudinal optical (LO) modes, a long-range electric field is generated that becomes macroscopic in the long-wavelength limit^13–15^. Creating an additional electric energy density in the material is more costly, and thus the frequency of the LO mode is blue-shifted. While the strength of this effect depends on the dielectric properties of the material (BECs and the high-frequency limit of the dielectric tensor ***ϵ***_*∞*_), its dependency on phonon momenta and size is ruled solely by dimensionality, and we argue here that this specific fingerprint on the dispersion relations can be exploited for spectroscopic characterization or in opto-electronic devices. In 3D, the energy shift of the LO mode is constant around the Brillouin zone center as a function of the norm of the momentum. At variance, in 2D it has been shown to depend linearly on momentum and to vanish at Γ exactly^3,4,16^. This breakdown can be expected in 1D systems as well^5,17–20^; nonetheless, its actual behavior remains an open question.

The dielectric contribution to the dispersion of the LO mode is often described in terms of a deviation from the transverse optical (TO) mode: LO-TO splitting. This is because in many materials (e.g., with cubic/tetragonal symmetries or planar hexagonal) LO and TO modes would be degenerate in the absence of dielectric effects^13,15,21–24^. However, the lifting of these degeneracies ultimately depends on the symmetries and the dimensionality of the crystal. In 3D, with 3 equivalent directions at most, optical modes are up to triply degenerate at the zone center (based on group theory considerations^25^). In 2D, these modes are up to doubly degenerate, while a splitting with respect to the out-of-plane optical (ZO) modes always persists since in- and out-of-plane displacements are nonequivalent. In 1D, the longitudinal direction is clearly different from the other two, possibly degenerate with each other. Thus, there is no degeneracy to recover, even if the polar energy shift vanishes. Accordingly, we will speak of dielectric or polar shift rather than LO-TO splitting.

In this work, we investigate infrared-active phonons, developing for 1D the analysis made^22–24^ for 3D^21^ and 2D materials^3,4^. We implement density-functional perturbation theory (DFPT)^22^ with 1D open-boundary conditions^26^ (see Supplementary Methods for further details) to capture the response of isolated 1D systems; we then derive an analytical model describing the interplay between the phonon-induced polarization and electronic screening. Notably, such model enables to interpret Raman and infrared spectra of 1D systems, thus greatly aiding material characterization at the nanoscale. We investigate the dispersion characteristics of prototypical systems such as BN atomic chains, BN nanotubes, and GaAs nanowires. Remarkably, we show the collapse of the dielectric shift at Γ and we derive its logarithmic asymptotic behavior as a function of phonon wavevector *q*_*z*_ and radius *t* of the material. This analysis allows to interpret and predict vibrational properties as a function of dimensionality, providing insights into the behaviour of nanomaterials (this includes nanotubes^5,17,18,27^, nanowires^16,19,28–30^, atomic chains and the major case of polymers^31–36^—systems widely discussed in the literature and central for nanotechnological applications) and paving the way to similar developments for optical and transport properties. As an example, we show how the radius of a 1D system could be extracted from infrared or Raman spectra by discussing the experimental results presented in ref. ^28^.

## Results and discussion

### Analytical model

We shall frame the discussion by introducing an electrostatic model for the electric field generated by LO phonons, and discuss its consequences on their dispersion relations. Here, the 1D system is described as a charge distribution periodic along the *z*-axis and homogeneous in the radial direction within an effective radius *t*, with vacuum outside. Within a dipolar approximation, the atomic displacement pattern ${{{{\bf{u}}}}}_{\nu }^{a}$ associated with a phonon *ν* of momentum ${{{\bf{q}}}}={q}_{z}\hat{{{{\bf{z}}}}}$ induces a polarization density

1 $${{{\bf{P}}}}({q}_{z})=\frac{{{{{\rm{e}}}}}^{2}}{L}\mathop{\sum}\limits_{a}{{{{\bf{Z}}}}}_{a}\cdot {{{{\bf{u}}}}}_{\nu }^{a}({q}_{z})\,,$$

where e is the unit charge, *L* is the unit-cell length, and **Z**_*a*_ is the BEC tensor for each atom *a* within the unit cell. The corresponding charge density **q** ⋅ **P**(*q*_*z*_) is the source of the electric field, termed Fröhlich due to the related electron-phonon interaction, and vanishes as soon as the phonon propagation (along momentum ${{{\bf{q}}}}={q}_{z}\hat{{{{\bf{z}}}}}$) and the polarization (along displacements ${{{{\bf{u}}}}}_{\nu }^{a}$) are orthogonal. Thus, within this model, only phonons labeled as LO in the long-wavelength limit generate a sizable electric field and experience the dielectric shift. In the following, we focus on strictly in-chain atomic displacements ${{{{\bf{u}}}}}_{\nu }^{a}\to {{{{\bf{u}}}}}_{{{{\rm{LO}}}}}^{a}$ and we assume BECs and the macroscopic dielectric tensor to be diagonal.

By solving the associated Poisson equation, we derive the new electrical forces and the resulting change to the phonon frequencies. The full derivation is reported in Supplementary Methods. After some manipulation, the general expression for a material in *n*-dimensions can be recast in the form

2 $${\omega }_{{{{\rm{LO}}}}}=\sqrt{{\omega }_{0}^{2}+{{\Delta }}{\omega }_{\max }^{2}\left[1-{{{\Delta }}}_{{{{\rm{nD}}}}}({{{\bf{q}}}},t)\right]}\,,$$

where *ω*_0_ is the reference value for the LO branch in the absence of any additional contribution from polarity. To ease the comparison between dimensionalities, we have highlighted two main contributions: ${{\Delta }}{\omega }_{\max }^{2}$ and Δ_nD_. The prefactor ${{\Delta }}{\omega }_{\max }^{2}=\frac{4{{{\rm{\pi }}}}{{{{\boldsymbol{e}}}}}^{2}}{{\epsilon }_{i}^{{{{\rm{m}}}}}{{\Omega }}}{\left({\sum }_{a}\frac{{{{{\bf{Z}}}}}_{a}\cdot {{{{\bf{e}}}}}_{{{{\rm{LO}}}}}^{a}}{\sqrt{{M}_{a}}}\right)}^{2}$ corresponds to the maximum value of the shift set by the dielectric properties of the nD crystal. Here, Ω is the volume of material in a cell, i.e., the volume of the unit cell in 3D, or the cell area times the thickness in 2D, or the cell length times the section in 1D. ${\epsilon }_{i = z}^{{{{\rm{m}}}}}$ is the dielectric tensor component for the propagation direction $\hat{{{{\bf{z}}}}}$ and ${{{{\bf{e}}}}}_{{{{\rm{LO}}}}}^{a}$ is the LO eigenvector for an atom *a* scaled by its mass *M*_*a*_. This prefactor is then modulated by Δ_nD_, whose expression depends on dimensionality: it is zero in 3D, while dependent on phonon momenta (in-plane or in-chain) and size *t* (thickness or radius) in 2D and 1D. The 1D fingerprint derived from the model presented here (assuming a diagonal and isotropic ***ϵ***_*∞*_, i.e., ${{{{\boldsymbol{\epsilon }}}}}^{{{{\rm{m}}}}}\to {\epsilon }_{1{{{\rm{D}}}}}{\mathbb{I}}$) reads

3 $${{{\Delta }}}_{1{{{\rm{D}}}}}({q}_{z},t)=2{I}_{1}(| {q}_{z}| t){K}_{1}(| {q}_{z}| t)\left(1-\frac{2{\epsilon }_{1{{{\rm{D}}}}}\sqrt{{{{\rm{\pi }}}}}{q}_{z}t{I}_{1}(| {q}_{z}| t){K}_{0}(| {q}_{z}| t)-{G}_{24}^{22}(| {q}_{z}{| }^{2}{t}^{2})}{2\sqrt{{{{\rm{\pi }}}}}{q}_{z}t({\epsilon }_{1{{{\rm{D}}}}}{I}_{1}(| {q}_{z}| t){K}_{0}(| {q}_{z}| t)+{I}_{0}(| {q}_{z}| t){K}_{1}(| {q}_{z}| t))}\right)\,,$$

where *I*_*n*_(*x*), *K*_*n*_(*x*) are the *n*^*th*^-order modified cylindrical Bessel functions, and $${G}_{pq}^{mn}\left(\left.\begin{array}{c}{a}_{1},...,{a}_{p}\\ {b}_{1},...,{b}_{q}\end{array}\right| x\right)$$ is the Meijer G-function. The limit behavior of Eq. (3) in the vicinity of Γ is ${{{\Delta }}}_{1{{{\rm{D}}}}}({q}_{z},t)=1-\frac{{q}_{z}^{2}{t}^{2}}{2}(C({\epsilon }_{1{{{\rm{D}}}}})-{\epsilon }_{1{{{\rm{D}}}}}\log ({q}_{z}t))$, where *C*(*ϵ*_1D_) is a constant (independent of *q*_*z*_*t*) and is reported in Supplementary Methods. Note that the equivalent in 2D would be^3^ ${{{\Delta }}}_{{{{\rm{2D}}}}}({{{{\bf{q}}}}}_{{{{\bf{p}}}}},t)=1-\frac{{\epsilon }_{{{{\rm{2D}}}}}t| {{{{\bf{q}}}}}_{p}| }{2+{\epsilon }_{{{{\rm{2D}}}}}t| {{{{\bf{q}}}}}_{{{{\bf{p}}}}}| }\,,$ leading in that case to a linear collapse of the dielectric shift in terms of in-plane phonon momentum **q**_*p*_.

Eqs. (2) and (3) are the central analytical result of this work. In particular, Δ_1D_ dictates the transition from a momentum-dependent (1D-like) to a momentum-independent (3D-like) shift, similar to what is observed in 2D^3^. In the *q*_*z*_*t* → 0 limit, Δ_1D_ → 1 and the shift breaks down at Γ with an overbending at small but finite *q*_*z*_^5,17–20^. Instead, if considering the opposite *q*_*z*_*t* → *∞* limit, the modulation typical of low-dimensionality vanishes and one is left with the well-known constant 3D shift. The reason behind this transition is intuitive. For small perturbing momenta (real-space long-range interactions), the electric-field lines associated with the polarization density spread far away in the surrounding medium, leading to vanishing dipolar interactions and shift: the material perceives itself as an infinitely thin 1D system surrounded by vacuum. As the momentum increases (short range in real space), these lines get more and more confined within the material and the dipole-dipole interactions eventually resemble those of a bulk material, insensitive to the boundaries.

As we will demonstrate, our model is in agreement with ab-initio results, particularly with respect to the *q*_*z*_ and *t* behaviors. However, while the splitting and BECs can be divided by the screening function *ϵ*(*q*_*z*_), the familiar form of this division^3,21^ is not immediately apparent in our model. To complement our description of the mechanism, we provide a simpler model in Supplementary Discussion, which is less accurate but easier to interpret.

### Application to chains, wires and tubes

We now combine our analytical findings with first-principles calculations. In this endeavour, DFPT^22^ represents a valuable ally, although some modifications are required when dealing with low dimensionality^3,4,9,37–39^. The major one stems from periodic-boundary conditions (PBCs) leading to spurious long-range Coulomb interactions between periodic images. When the electronic charge density is perturbed at momentum *q*, the reach of these interactions scales as *λ* = 2π/*q* in the out-of-chain (or plane) directions. It follows that, for long-wavelength perturbations, these cross-talks persist even for very large distances, turning the response of the isolated 1D system into the one of a fictitious 3D system of periodic replicas. For a systematic and physical solution to this issue, we have implemented a 1D Coulomb cutoff technique, based on the version proposed in ref. ^38^, in the relevant packages (PWScf, PHonon) of the Quantum ESPRESSO distribution^22,40,41^. This implementation is summarized in Supplementary Methods while fully detailed in an upcoming publication^26^, and restores the physical open-boundary conditions for the computation of total energies, forces, stress tensors, phonons, and electron-phonon interactions.

**Fig. 1:**
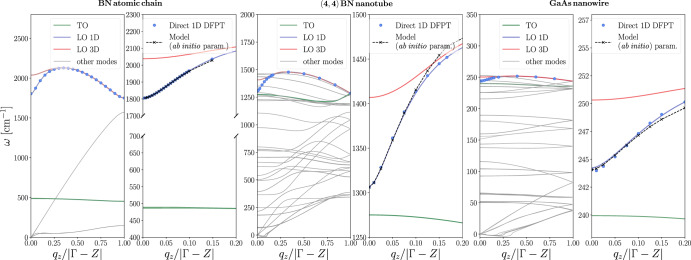
Phonon dispersion of BN atomic-chain, (4,4) nanotube, and wurtzite GaAs nanowire of 24 atoms including the hydrogen atoms saturating the surface dangling bonds. For each material, left panel compares 3D-PBC and 1D-OBC DFPT calculations, explicit for 1D (symbols) and interpolated for both (lines). Right panel shows the agreement of our model with 1D DFPT for the LO branch in the long-wavelength limit.

In the following, we focus on BN atomic chains, BN armchair nanotubes, and GaAs nanowires. The scope is threefold: to show the relevance of the open-boundary conditions for linear response in 1D, to validate Eq. (3), and to discuss the underlying physics and the transition between dimensionalities. The details of the calculations and model parametrization (also independently obtained via 1D DFPT) are given in Supplementary Methods. A crucial parameter in our investigation is the radius *t*, which determines the radial extent of both the polarization density and the electronic charge density. To determine this parameter, we examine the radial electronic charge density profile, which is averaged on cross-sectional planes along the 1D-axis, and set a meaningful threshold for each system. For each material we compute the phonon dispersions and we plot it in full in the left panel, zooming around the LO-TO splitting in the right panel (see Fig. 1). We focus on the modes similar to those of the bulk 3D or 2D parents, identifying the purest longitudinal mode (hLO), highest in energy and associated to the largest polarization density, as well as the corresponding tangential or bulk-like transverse modes. For clarity, colors are meant to highlight these phonon branches, while the others are left in gray in the background. Note that these materials are dynamically stable, see Supplementary Methods for a discussion concerning the long-wavelength dispersion of acoustic phonons.

Figure 1 shows the effect of the spurious interactions between periodic images. In 3D DFPT (i.e., 3D PBCs), we always recover a rather flat LO branch (red) with a finite dielectric shift at Γ, which possibly adds up to the splitting with respect to TO phonons (green) due to crystal symmetries and dimensionality. This is the 3D response of an array of interacting 1D materials. On the contrary, with 1D DFPT (i.e., 1D open-boundary conditions), the amount of energy built by LO phonons (blue) is shown to vanish at the zone center and the branch exhibits a logarithmic overbending in the long-wavelength limit: this is the response of the isolated 1D material given by Eq. (3). The correction introduced by the Coulomb cutoff is shown to be significant for small *q*_*z*_, where low-dimensionality comes into play. The Brillouin zone range over which the discrepancy between 3D and 1D DFPT extends is determined by the amount of vacuum in the simulation cell. In PBCs, the larger the vacuum, the smaller the region affected by the stray fields, and the softer the LO branch; this latter asymptotically converges to the 1D limit. The true physical behavior is fully recovered only in the presence of the cutoff, since for momenta smaller than the inverse of the distance between periodic images there will always be the response of a 3D periodic system, i.e., a non vanishing polar shift.

In the right panels of Fig. 1 we present the comparison between 1D DFPT and the analytical model we have derived. For all materials, a good agreement is found at the very least within the first 10–20% of the Brillouin zone, that is the long-wavelength limit targeted by the model. The strength of the effect, being the range of frequencies over which the overbending extends, is material dependent and ruled by the square of the screened effective charges, i.e., $\frac{{{{{\bf{Z}}}}}_{a}^{2}}{{\epsilon }_{1{{{\rm{D}}}}}}$ (see Eq. (2)). By comparing the materials in Fig. 1, the polar shift is obviously most pronounced in BN: around 200 cm^−1^ in the case of nanotubes, consistently with 2D and 3D hexagonal BN^3,42,43^, and around 400 cm^−1^ for the chain. In the BN chain, the larger increase is due to the crystal structure differing from the hexagonal one common to the other allotropes. The effect is more subtle in GaAs, of about 10 cm^−1^, because of the significantly smaller BECs. The strength of the shift is estimated based on the blue curve only (1D OBCs) and by considering the difference between the maximum frequency (i.e., *q*_*z*_ ≈ 25% of the Brillouin zone, where the transition towards a 3D regime happens) with respect to the Γ value (i.e., 1D null shift). Note that as a consequence of the pseudopotentials used in this work, the GaAs frequencies of the nanowire are strongly underestimated. The actual range of the polar effect should be approximately 20 cm^−1^. However, here the focus is mainly on the qualitative trend of the polar effect more than the exact frequencies. Different pseudopotentials are instead used for bulk GaAs to compare with experiments in the following section and these lead to phonon frequencies in agreement with the literature.

In low-dimensional materials, as a consequence of the vanishing polar shift, the remaining LO-TO splitting at the zone center is purely ‘mechanical’, i.e., due to structurally different atomic displacements because of symmetry and dimensionality. Among the selected materials, the chain represents the ultimate 1D system and exhibits the largest mechanical splitting (i.e., larger asymmetry between displacement directions). Instead, nanotubes and nanowires sit in between 1D and 2D and 3D, respectively, as a function of their diameter. The mechanical splitting at Γ is expected to decrease as *t* → *∞*, converging to the 2D or 3D case. Similarly, the polar shift asymptotically converges to its higher-dimensional limit. Here, ‘asymptotically’ is key, since the 1D nature of the material will always suppress the polarity-induced electric field at small enough momenta. Thus, the effect of the radius increase is visible in the long-wavelength regime: the range of momenta over which the shift vanishes shrinks and the discontinuity at Γ due to direction-dependent BECs is transferred from the prefactor of the logarithmic overbending (1D) to the slope^3^ (2D) or the value^13,15,21–24^ (3D) of the polar shift.

**Fig. 2:**
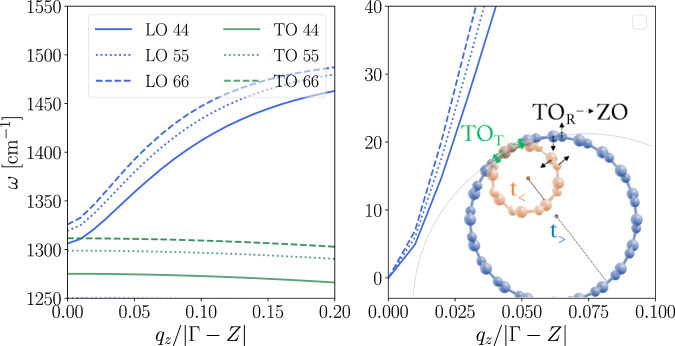
Size effects (mechanical and polar) on LO and TO modes for BN (6,6), (5,5) and (4,4) armchair nanotubes from 1D-DFPT. Left panel compares both modes. Right panel focuses on the polar shift for the LO branch by setting as common offset *ω*_0_ = 0. The sketch represents the mechanical evolution of tangential TO_T_ vs radial TO_R_ optical modes.

Focusing on nanotubes, Fig. 2 compares the first-principles results for (4,4), (5,5) and (6,6) BN tubes. In the right panel, decreasing the curvature is shown to stiffen the logarithmic LO behavior and approach the linear signature of 2D materials^3^. The left panel shows instead the absolute values of the optical frequencies for each tube and focuses on the mechanical size effects. There, one can observe two trends. On one hand, it is known throughout the literature that increasing the radius mechanically blue-shifts both TO and LO phonons at zone center^6,16–19,27,29,30,44–48^. On the other hand, the same radius increase progressively reduces the mechanical splitting, converging towards a finite or null gap depending on the symmetries of the 2D parent (as sketched in Fig. 2). Note that the mechanical shifting is much stronger for the TO mode, which can be understood by considering that the atoms are displaced in the non-periodic direction. Furthermore, since the mechanical contribution to the LO shift actually opens the LO-TO gap (pushing LO up), the closing of the gap can be attributed to the stronger increase of the TO frequency. A similar analysis holds for nanowires as well, but in this case the transition is from 1D to 3D. The mechanical effects are shared with nanotubes, depending only on crystal symmetries. As regards the dielectric shift, the range of momenta over which the splitting goes from zero to its constant 3D value progressively shrinks around Γ, the dispersion becoming progressively steeper and only asymptotically approaching the well-known discontinuity in the bulk.

### Raman and infrared characterization

Once the relevant parameters in Eqs. (2), (3) are known, empirically or from first-principles, the model unveils a one-to-one relationship between first-order LO Raman/infrared lines (i.e., frequency) and radius *t*. This makes the model a very valuable tool for spectroscopic characterization of 1D systems. The phonon probed in experiments is with small but finite momentum *q*_*z*_, related to the laser wavelength *λ* by momentum conservation law (*q*_*z*_ = 2π/*λ*). Thus, the larger the *λ*, the closer to Γ is the *q*_*z*_ probed. The evolution with size of the hLO branch is given by Eq. (2), where size effects are conveyed by the radius explicitly appearing in the Δ_1D_(*q*_*z*_, *t*, *ϵ*_1D_). In passing, note that the dielectric properties and the absolute positions of LO/TO modes may actually change with size due to mechanical reasons, as well as the q-dependency of the eigenvectors (see Supplementary Discussion). We emphasize that the roles of *t* and *q*_*z*_ in Δ_1D_ are symmetric (i.e., Δ_1D_ depends on the product *q*_*z*_*t*). Thus, the behavior of the frequency versus size *ω*_LO_(*t*) at fixed phonon momenta is the same as the phonon dispersion *ω*_LO_(*q*_*z*_) at fixed radii. The polar shift increases logarithmically at small *t*, then approaches a maximum set by the bulk splitting. The increase is sharper for smaller wavelengths, meaning that a larger *λ* would instead ease size resolution.

Raman/infrared experiments on single, isolated and semiconducting wires/tubes are mostly missing, and closest to the conditions discussed here is the work of ref. ^28^, where the authors propose a strategy to grow ultrathin GaAs nanoneedles with atomically sharp tips (*t* ≈ 10 nm, mostly wurtzite) on top of thicker bases (*t* ≈ 100 nm, zincblende). The nanowires are arranged in regular arrays with a spacing of 1000 nm. This distance, compared with the laser spot, is large enough to avoid significant inter-wire cross-talks hindering the response of the single wire. Then, size effects are investigated with room-temperature Raman spectroscopy by pointing a semiconducting laser probe at 785 nm on either the tip or the base. The observed spectra are composed of two peaks each. For the base, the TO and LO modes are found at 268 cm^−1^ and 285 cm^−1^ (LO-TO splitting ≈ 17 cm^−1^), respectively. The tip spectrum, instead, systematically exhibits, besides broadening, a stable TO mode and a down shift of about 3 cm^−1^ for the LO mode. The observed change in the LO-TO separation, switching from the base to the tip, appears to be almost entirely due to the change in LO position. A mechanical red shift is expected to affect equally the two modes or mostly the TO, which is instead stable. This points to the dielectric nature of the phenomenon, i.e., the vanishing polar shift and its size dependence.

**Fig. 3:**
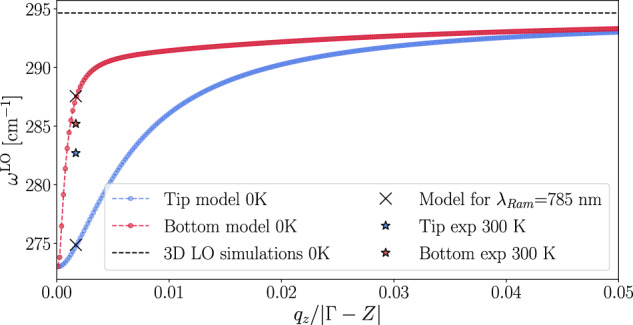
Evolution of the LO branch in the long-wavelength limit for the two GaAs nanowires from ref. ^28^ (10 and 100 nm in radius) obtained by the model. The comparison with experiments^28^ is reported.

In Fig. 3 the hLO mode for the two nanowires as given by the present model is compared with the data from ref. ^28^. We assume both systems to be large enough that we can parameterize (ab-initio at *T* = 0 K) Eq. (2) using the bulk ***ϵ***^m^ and BECs, and purely longitudinal and constant eigenvectors. This results in an upper limit in terms of frequencies for each *q*_*z*_ and *t*. Shrinking the size corresponds to a decrease in the branch steepness close to Γ and to the observed blue shift. The agreement between experiments and the model is very good for the thicker base of the nanowire and semiquantitative with respect to the measured blue shift for the tip. There are multiple reasons behind this, such as temperature effects and mechanical contributions (not accounted for in this parametrization), but especially the uncertainty on the phonon momenta probed experimentally. However, the most reasonable explanation is that base and tip signals are not fully decoupled. Thus, experiments probe a mixture of base and tip phonons and fall in between theoretical predictions for the two nanowires’ size. In this regards, further comparison with experiments on single-wires/tubes with constant radius will serve to validate the details of the proposed model. We hope this paper will motivate future work in this direction.

In conclusion, we have argued for a breakdown of the dielectric shift experienced by LO phonons in 1D systems, and shown its exact asymptotic behavior in terms of phonon momenta and material size. This understanding and the accurate first-principles description provided by the open-boundary conditions implementation of this work represent a transparent and insightful advance in the field. First, the model can be exploited by the experimental community to aid material characterization of 1D materials. In addition, the proposed computational framework unlocks the full potential of DFPT for 1D system, paving the way to further studies which go beyond vibrational properties and may revolve around electron-phonon coupling, and optical properties.

## Methods

In this section we report the computational details relevant for this work. Then, we summarize the key assumptions in the derivation of the analytical model and its parametrization. For more details on each of these topics, see Supplementary Methods.

### Computational details

First-principles calculations of structural properties and phonons are performed with the Quantum ESPRESSO package^22,40,41^, by combining DFT and DFPT. We use the PBE exchange-correlation functionals for all materials, with the exception of bulk wurtzite GaAs for which we use norm-conserving pseudopotentials within the local density approximation from the Original QE PP Library. For 1D-DFT/DFPT calculations, 1D periodic-boundary conditions (i.e., the 1D cutoff and the 1D phonon Fourier-interpolation based on the analytical model) are applied to properly describe linear response to a phonon perturbation^26^. The pseudopotentials (except for the ones used for bulk GaAs) are taken from the Standard Solid-State Pseudopotentials (SSSP) library (precision version 1.1)^49^ and the wave-function and charge density energy cutoff have been selected accordingly: 110 and 440 Ry for the chain, 80 and 440 Ry for nanotubes, and 90 and 720 Ry for the GaAs nanowire. For bulk GaAs, we selected instead a wave-function cutoff of 80 Ry. We treated all the materials under study as non-magnetic insulator (i.e., fixed occupations) and a fine electron-momenta distance of approximately 0.2 Å^−1^ (unshifted mesh) has been used to sample the Brillouin zone. The convergence of all the relevant parameters have been performed aiming to an accuracy on the final phonon frequencies of few cm^−1^.

### Analytical model and its parametrization

The 1D material is modeled by assuming a distribution of charge periodic along the *z*-axis and homogeneous in the radial direction **r**_**⊥**_ within an effective radius *t*. We assume vacuum outside, i.e., free standing materials, but the generalization to include the effects from other surrounding dielectric media is straightforward. The dielectric properties of the 1D system are then modeled as diagonal BECs and macroscopic dielectric tensors, isotropic only in the two out-of chain directions:

$${{{{\boldsymbol{\epsilon }}}}}^{{{{\rm{m}}}}}=\left(\begin{array}{lll}{\epsilon }_{\perp }^{{{{\rm{m}}}}}&0&0\\ 0&{\epsilon }_{\perp }^{{{{\rm{m}}}}}&0\\ 0&0&{\epsilon }_{z}^{{{{\rm{m}}}}}\\ \end{array}\right){{{{\bf{Z}}}}}_{a}=\left(\begin{array}{lll}{Z}_{a,\perp }&0&0\\ 0&{Z}_{a,\perp }&0\\ 0&0&{Z}_{a,z}\\ \end{array}\right)\,.$$

The derivation of the phonon frequencies is based on the dipolar approximation, i.e., the polarization density **P**(**q**) is written in terms of the BECs only, neglecting higher orders contribution in the effective charges (i.e., quadrupoles, octupoles,...)^4,50^. Then, we solve the electrostatic problem associated to this polarization density, exploiting the periodicity of the system and accounting for its dimensionality. The solution of the associated Poisson equation is fully analytical. Once we have the full formula for *ω*_LO_(*q*_*z*_, *t*), we simplify it using isotropic dielectric tensors, reducing the tensor to a scalar quantity: ${{{{\boldsymbol{\epsilon }}}}}^{{{{\rm{m}}}}}\to {\epsilon }_{1{{{\rm{D}}}}}{\mathbb{I}}$. This approximation may appear drastic but it is effective for our scopes and for all the systems discussed in this work. For more details and in-depth discussion, see Supplementary Methods.

The analytic results in Fig. 1 rely on the ab-initio parameters obtained independently via DFPT in 1D open-boundary conditions. Equation (2) involves several physical quantities. Masses, eigenvectors, eigenvalues and BECs are directly obtained from the underlying DFT and DFPT calculations. The only exceptions are the effective radius *t* and the dielectric tensor, that is its in-chain component *ϵ*_1D_ within our isotropic assumption. The 1D dielectric tensor in our model differs from the one computed in QE, ***ϵ***^QE^, which strongly depends on the size of the simulation cell. They are related via effective medium theory^9,51^ and the physical link is the polarizability. In our parametrization (see Supplementary Methods) we use:

4 $${\epsilon }_{1{{{\rm{D}}}}}=\frac{{c}^{2}}{{{{\rm{\pi }}}}{t}^{2}}({\epsilon }_{z}^{{{{\rm{QE}}}}}-1)\,,$$

where *c* is the out-of-chain length characterizing the supercell geometry, assumed to be the same in the x and y directions. Finally, a reasonable choice is to extract *t* from the radial electronic charge density profile averaged on the cross-sectional planes along the 1D-axis. This strategy has been applied to all the materials shown in Fig. 1 to compare with first-principles results. In order to interpret the experimental results in ref. ^28^, we adopt similar choices for the parametrization of the model. In this case, simulating from first-principles the two nanowires is prohibitively costly from a computational point of view because of their thickness. At the same time, thanks to the same thickness, their dielectric properties and the *ω*_0_ are expected to be similar to bulk GaAs. Thus, we extract all the needed quantities from simulations of bulk wurtzite GaAs, except for the *t* which is given directly in ref. ^28^ and the eigenvectors which are chosen to be constant and purely longitudinal.

### Supplementary information

Supplementary Information Infrared-active phonons in one-dimensional materials and their spectroscopic signatures

## Data Availability

All relevant computational results and data are provided in the Materials Cloud repository^52^.

## Code availability

DFT and DFPT calculations with 3D PBCs were performed with the open-source code QUANTUM ESPRESSO^40^, while the modification of the standard code to include 1D open-boundary conditions will be available at https://github.com/normarivano, pending the merge into the official branch, and detailed in ref. ^26^. The open-source code will be released after acceptance of the manuscript.
