# Supplementary material for: Infrared-active phonons in one-dimensional materials and their spectroscopic signatures
Source: NPJ Comput Mater. 2023 Oct 18;9(1):194. doi: 10.1038/s41524-023-01140-2 (PMC11041644; doi:10.1038/s41524-023-01140-2)
Supplement: Supplementary file 1 — Supplementary Information Infrared-active phonons in one-dimensional materials and their spectroscopic signatures [file 41524_2023_1140_MOESM1_ESM.pdf]

Supplementary Information

Infrared-active phonons in one-dimensional materials and their spectroscopic signatures

Norma Rivano,[1,*] Nicola Marzari,[1,2] and Thibault Sohier[3]

[1] *Theory and Simulations of Materials (THEOS), École Polytechnique Fédérale de Lausanne, CH-1015 Lausanne, Switzerland and National Centre for Computational Design and Discovery of Novel Materials (MARVEL), École Polytechnique Fédérale de Lausanne, CH-1015 Lausanne, Switzerland*

[2] *Laboratory for Materials Simulations, Paul Scherrer Institut, 5232 Villigen PSI, Switzerland*

[3] *Laboratoire Charles Coulomb (L2C), Université de Montpellier, CNRS, Montpellier, France*

[*] Corresponding author: nrivano@g.harvard.edu; Present address: John A. Paulson School of Engineering and Applied Sciences, Harvard University, 29 Oxford Street, Cambridge, Massachusetts 02138, USA.

I. SUPPLEMENTARY METHODS

In this section, we detail the analytical derivation whose results are summarized in the main text. We model the 1D material (see Supplementary Figure 1) by assuming a periodic charge distribution along the z-axis and an homogeneous charge distribution in the radial direction, confined within an effective radius t. We assume vacuum outside, but the generalization to other surrounding media is straightforward.

$$\boldsymbol{\epsilon}^{\mathrm{m}} = \begin{pmatrix} \epsilon_{\perp}^{m} & 0 & 0 \\ 0 & \epsilon_{\perp}^{\mathrm{m}} & 0 \\ 0 & 0 & \epsilon_{z}^{\mathrm{m}} \end{pmatrix} \mathbf{Z}_a = \begin{pmatrix} Z_{a,\perp} & 0 & 0 \\ 0 & Z_{a,\perp} & 0 \\ 0 & 0 & Z_{a,z} \end{pmatrix} .$$

Exploiting the periodicity along the z-axis, the longitudinal optical (LO) atomic displacement pattern can be decomposed as a superposition of phonon waves of momenta q_z:

$$\mathbf{u}_{\mathrm{LO}}^{a}(\mathbf{r}_{\perp}, z) = \sum_{q_z} \mathbf{u}_{\mathrm{LO}}^{a}(q_z) e^{izq_z} , \tag{1}$$

where $\mathbf{u}_{\mathrm{LO}}^{a} = \mathbf{e}_{\mathrm{LO}}^{a}/\sqrt{M_a}$ is the displacement of atom a given by the eigenvectors of the dynamical matrix $\{e_{\mathrm{LO}}^{a}(q_z)\}$ scaled by the nuclear masses M_a. This generates the following Fourier-transformed polarization density:

$$\mathbf{P}(\mathbf{r}_{\perp}, q_z) = \frac{\mathrm{e}^2}{L} \sum_{a} \mathbf{Z_a} \cdot \mathbf{u}_{\mathrm{LO}}^{a}(q_z) f(\mathbf{r}_{\perp}) , \tag{2}$$

where e is the unit charge, L is the unit-cell length in the periodic direction, $\mathbf{Z}_a$ is the tensor of Born effective charges (BECs) associated to each atom a, and we model the out-of-chain profile as a Heaviside step function in the radial coordinates, $\mathbf{r}_{\perp}$, $f(\mathbf{r}_{\perp}) = \frac{\theta(t-|\mathbf{r}_{\perp}|)}{\pi t^2}$ normalized to unity over the cross-sectional area ($\int_0^{\infty} f(r_{\perp}) 2\pi r_{\perp} \, dr_{\perp} = 1$). Note that the same effective radius t is used to define the extent of the dielectric medium (ions and electrons) and the polarization density. The induced Fröhlich potential, V_{Fr}, (i.e., the same one related to the well-known electron-phonon coupling) is obtained by solving the Poisson equation:

$$\boldsymbol{\nabla} \cdot [\boldsymbol{\epsilon} \boldsymbol{\nabla} V_{\mathrm{Fr}}(\mathbf{r})] = -4 \boldsymbol{\nabla} \cdot \mathbf{P}(\mathbf{r}) , \tag{3}$$

where the dielectric tensor, $\boldsymbol{\epsilon}$, is $\boldsymbol{\epsilon}^{\mathrm{m}}$ inside the material, while outside it reduces to the vacuum dielectric constant, i.e., $\epsilon_0 = 1$. The right-hand side of Eq. 3 Fourier-transforms as $q_z \cdot P(q_z)$. The potential in Eq. 3 needs to fulfill the proper electrostatic boundary conditions (i.e., continuity of the potential and its derivatives at the interfaces, and no divergences):

1. Continuity of the parallel electric field $E_{\parallel}$ (from Maxwell's equation $\boldsymbol{\nabla} \times \mathbf{E} = 0$);

2. Continuity of the orthogonal dielectric displacement $D_{\perp}$ (from Maxwell's equation $\boldsymbol{\nabla} \cdot \mathbf{D} = 0$);

3. No divergences in V_{Fr};

4. Continuity of V_{Fr} ,

where parallel ($\|$) and orthogonal ($\perp$) are defined with respect to the chain axis. It is worth noticing that the continuity of $E_{\parallel}$ guarantees also the potential to be continuous. At this point, our problem reduces to solve a second order non-homogeneous linear partial differential equation. The most general solution of the potential can thus be expressed as sum of the homogeneous solution (V_{h}) and a particular one (V_{p})

$$V_{\mathrm{Fr}} = V_{\mathrm{p}} + V_{\mathrm{h}}\,, \tag{4}$$

where V_{h} actually corresponds to a family of possible solutions of the homogeneous equation defined up to one or more constants, to be determined by enforcing the desired boundary conditions. Note that it is the complete solution V_{Fr} which has to satisfy the boundary conditions enumerated above, not the single V_{p} and V_{h}. Thus, we write the LO atomic displacement pattern as a superposition of phonon waves of momenta q_z:

$$\mathbf{P}(\mathbf{r}_{\perp}, z) = \frac{\mathrm{e}^2}{L}\sum_a \mathbf{Z}_a \cdot \mathbf{u}^a(\mathbf{r}_{\perp}, z) = \frac{\mathrm{e}^2}{L}\sum_a \sum_{q_z} \frac{\mathbf{Z}_a \mathbf{e}^a(q_z)}{\sqrt{2M_a \omega_{q_z \mathrm{LO}}}} e^{izq_z} f(\mathbf{r}_{\perp})\,. \tag{5}$$

Similarly, we write down $V_{\mathrm{p}}(\mathbf{r}_{\perp}, z)$ by exploiting the periodicity along the z-axis and the radial symmetry:

$$V_{\mathrm{p}}(\mathbf{r}_{\perp}, z) = \int_{R^2} \sum_{\mathbf{q}_z} V_{\mathrm{p}}(\mathbf{q}_{\perp}, q_z) e^{i\mathbf{q}_{\perp}\cdot\mathbf{r}_{\perp}} e^{iq_z z}\, d\mathbf{q}_{\perp}\,. \tag{6}$$

We then substitute Eqs. 5 and 6 in Eq. 3 and we obtain the particular solution inside the cylinder (i.e., the 1D system)

$$V_{\mathrm{p}}(\mathbf{q}_{\perp}, q_z) = \frac{2\mathrm{e}^2}{\pi t L} \frac{J_1(t|\mathbf{q}_{\perp}|)}{|\mathbf{q}_{\perp}|} \frac{\sum_a (i\mathbf{q}\mathbf{Z}_a \cdot \mathbf{e}^a_{\mathrm{LO}}(q_z))/\sqrt{2M_a \omega_{q_z \mathrm{LO}}}}{(\epsilon_{\perp}\,|\mathbf{q}_{\perp}|^2 + \epsilon_z\,|\mathbf{q}_z|^2)}\,. \tag{7}$$

Outside the cylinder, we can simply choose a solution of the corresponding equation, that is $\nabla^2 V_{\mathrm{p}}(\mathbf{r}_p, z) = 0$, such that the out-of-chain component of the electric field is continuous

at the interface. It is now possible to proceed to find the homogeneous solution V_{h}. This solution has to solve the homogeneous Poisson equation:

$$\boldsymbol{\nabla} \cdot [\boldsymbol{\epsilon} \boldsymbol{\nabla} V_{\mathrm{h}}(\mathbf{r})] = 0\,. \tag{8}$$

Once again, exploiting the periodicity of the potential along the 1D axis, we can write:

$$V_{\mathrm{h}}(\mathbf{r}_\perp, z) = \sum_{q_z} V_{\mathrm{h}}(\mathbf{r}_\perp, q_z) e^{izq_z}\,. \tag{9}$$

Substituting into the homogeneous equation written before, we obtain:

$$\frac{\partial^2 V_{\mathrm{h}}(\mathbf{r}_\perp, q_z)}{\partial r_\perp^2} + \frac{1}{r_\perp}\frac{\partial V_{\mathrm{h}}(\mathbf{r}_\perp, q_z)}{\partial r_\perp} = \begin{cases} \frac{\epsilon_z}{\epsilon_\perp} \left|\mathbf{q}_z\right|^2 V_{\mathrm{h}}(\mathbf{r}_\perp, q_z) & if\, |\mathbf{r}_\perp| < t \\ \left|\mathbf{q}_z\right|^2 V_{\mathrm{h}}(\mathbf{r}_\perp, q_z) & if\, |\mathbf{r}_\perp| > t\,, \end{cases}$$

and the solution is:

$$V_{\mathrm{h}}(\mathbf{r}_\perp, q_z) = \begin{cases} c_1 J_0\Big(i\sqrt{\tfrac{\epsilon_z}{\epsilon_\perp}} r_\perp q_z\Big) + c_2 Y_0\Big(-i\sqrt{\tfrac{\epsilon_z}{\epsilon_\perp}} r_\perp q_z\Big) & if\, |\mathbf{r}_\perp| < t \\ c_3 J_0(i r_\perp q_z) + c_4 Y_0(-i r_\perp q_z) & if\, |\mathbf{r}_\perp| > t\,. \end{cases} \tag{10}$$

We have now four different constants which we can fix in order to fulfill the electrostatic boundary conditions. After imposing the continuity conditions, we find the potential

$$V_{\mathrm{Fr}}(q_z) = \frac{4\mathrm{e}^2}{\epsilon_\perp t^2 L q_z} \sum_a \frac{\mathbf{Z}_a \cdot \mathbf{e}^a_{\mathrm{LO}}(q_z)}{\sqrt{2M_a \omega_{q_z\mathrm{LO}}}} \Big[1 - 2I_1(q_z t)K_1(q_z t)\Big(1 - \frac{2\epsilon_\perp\sqrt{\pi} q_z t I_1(q_z t)K_0(q_z t) - G^{22}_{24}(q_z^2 t^2)}{2\sqrt{\pi} q_z t(\epsilon_\perp I_1(q_z t)K_0(q_z t) + I_0(q_z t)K_1(q_z t))}\Big)\Big]\,, \tag{11}$$

named V_{Fr} to highlight that this term is the one describing the interaction between LO phonons and electrons and thus leading to the well-known electron-phonon coupling. For the sake of simplicity, in Eq. 11 we used an isotropic assumption on the already diagonal dielectric tensor ($\boldsymbol{\epsilon}^{\mathrm{m}} \to \epsilon^{\mathrm{m}}_z \mathbb{I} = \epsilon_{\mathrm{1D}} \mathbb{I}$). We obtain the Coulomb screened interaction as

$$W_{\mathrm{c}}(q_z) = \frac{V_{\mathrm{Fr}}(q_z)}{q_z P_z(q_z)}\,. \tag{12}$$

We then derive the corresponding electric field $\mathbf{E}$ and the forces $\mathbf{F}_a$ acting on each ion a, being respectively:

$$\mathbf{E}(\mathbf{q}_z) = -\nabla V_{\mathrm{Fr}}(\mathbf{q}_z) = -W_{\mathrm{c}}(\mathbf{q}_z)(\mathbf{q}_z \cdot \mathbf{P}(\mathbf{q}_z))\mathbf{q}_z\,, \tag{13}$$

$$\mathbf{F}_a = \mathbf{E} \cdot \mathbf{Z}_a = -W_{\mathrm{c}}(\mathbf{q}_z)\Big(\mathbf{q}_z \cdot \frac{\mathrm{e}^2}{L} \sum_{a'} \mathbf{Z}_{a'} \cdot \mathbf{u}^{a'}_\nu\Big)(\mathbf{q}_z \cdot \mathbf{Z}_a)\,. \tag{14}$$

These new forces eventually turn into an additional contribution to the dynamical matrix:

$$D_{ai,bj}(q_z) = -\frac{\mathrm{e}^2}{L} W_c(\mathbf{q}_z) \frac{(\mathbf{q}_z \cdot \mathbf{Z}_a)_i (\mathbf{q}_z \cdot \mathbf{Z}_b)_j}{\sqrt{M_a M_b}}\,, \tag{15}$$

where a and b label the atoms, while i and j index the cartesian coordinates. This additional contribution is reflected in the eigen-solution of the dynamical matrix. Finally we obtain the phonon frequencies for the LO mode:

$$\omega_{\mathrm{LO}}(q_z, t) = \sqrt{\omega_0^2 + W_{\mathrm{c}}(\mathbf{q}_z) \frac{\mathrm{e}^2 |q_z|^2}{L} \Big(\sum_a \frac{\mathbf{Z}_a \cdot \mathbf{e}^a_{\mathrm{LO}}}{\sqrt{M_a}} \Big)^2} = \sqrt{\omega_0^2 + \Delta\omega^2_{\mathrm{max}} \Big[1 - \Delta_{\mathrm{1D}}(q_z, t) \Big]} \tag{16}$$

where the 3D prefactor $\Delta\omega_{\mathrm{max}}$ and the dimensionality modulation $\Delta_{\mathrm{1D}}(q_z, t)$ specific of 1D are the ones reported in the main text:

$$\Delta\omega^2_{\mathrm{max}} = \frac{4\pi \mathrm{e}^2}{\epsilon_{\mathrm{1D}} \Omega} \Big(\sum_a \frac{\mathbf{Z}_a \cdot \mathbf{e}^a_{\mathrm{LO}}}{\sqrt{M_a}} \Big)^2, \tag{17}$$

$$\Delta_{\mathrm{1D}}(q_z, t) = 2 I_1(|q_z| t) K_1(|q_z| t) \Big(1 - \frac{2\epsilon_{\mathrm{1D}} \sqrt{\pi} q_z t I_1(|q_z| t) K_0(|q_z| t) - G^{22}_{24}(|q_z|^2 t^2)}{2\sqrt{\pi} q_z t (\epsilon_{\mathrm{1D}} I_1(|q_z| t) K_0(|q_z| t) + I_0(|q_z| t) K_1(|q_z| t))} \Big)\,. \tag{18}$$

Here we mention the limit behaviors. By Taylor expanding $\Delta_{\mathrm{1D}}(q_z, t)$ of Eq. 16 in the vicinity of Γ, we can extract the mentioned logarithmic overbending $q_z^2 t^2 \log(q_z t)$, an the 3D limit (i.e., $q_z t \to \infty$):

$$\Delta_{\mathrm{1D}}(q_z, t) = \begin{cases} 1 - \frac{q_z^2 t^2}{2} (C(\epsilon_{\mathrm{1D}}) - \epsilon_{\mathrm{1D}} \log(q_z t)) & \text{for } q_z t \to 0 \\ 0 & \text{for } q_z t \to \infty \end{cases} \tag{19}$$

where $C(\epsilon_{\mathrm{1D}})$ is constant with respect to $q_z t$ but it depends on the dielectric tensor and is equal to $-\frac{3}{4} + \frac{\gamma}{2}(1 - 2\epsilon_{\mathrm{1D}}) + \frac{\psi(3/2)}{2} + \log(2)(\epsilon_{\mathrm{1D}} + 1)$, with ψ being the digamma function. The transition between the two limit behaviors is somehow more complex with respect to what shown in 2D [1], with $(q_z t)_{\mathrm{critical}} \propto \frac{\mathrm{const.}}{\epsilon_{\mathrm{1D}}}$.

1D open-boundary conditions in density-functional perturbation theory: Phonon calculations reported in the main text are performed with density-functional perturbation theory (DFPT) in a newly implemented one-dimensional flavour. Using standard DFPT in 3D periodic-boundary conditions (PBCs) issues arise for the LO dispersion at small but finite momenta due to spurious long-range interactions between the periodic images. On the contrary, the computation exactly at Γ happens to be correct in 1D, as in 2D,

since the corresponding $\mathbf{q} = 0$ macroscopic field is ill-defined and absent from the simulation (i.e., zero dielectric shift). The standard method of simply increasing the vacuum between images only reduces the affected portion of the Brillouin zone, while the computational cost significantly increases. Thus, we propose to properly address this issue via the Coulomb cutoff technique [2–5]. Our version is based on the one proposed in Ref. [3], is implemented in the relevant packages (PWScf and PHONONS) of the Quantum ESPRESSO distribution (QE) [6–8] and soon to be released [9]. This implementation leads to the correct 1D boundary conditions for the computation of total energies, forces, stress tensors, phonons, and the electron-phonon interaction. A further in-depth publication will follow detailing the implementation and fully documenting its relevance in terms of long-ranged physics in 1D systems [9]. Here, we report in a nutshell the main idea, sketched in Supplementary Figure 2. The core strategy consists in redefining the Coulomb kernel $v_{\mathrm{c}} \to v_{\mathrm{c}}^*$ as:

$$v_{\mathrm{c}}^*(\mathbf{r}) = \frac{\theta(l_{\mathrm{c}} - |\mathbf{r}_\perp|)}{|\mathbf{r}|} \,. \tag{20}$$

All the relevant potentials are then obtained as convolution of this kernel with the charge density

$$V(\mathbf{r}) = e \int \rho(\mathbf{r}') v_{\mathrm{c}}^*(|\mathbf{r} - \mathbf{r}'|) \, d\mathbf{r}' \,, \tag{21}$$

in such a way that a given charge in the 1D system interacts only with charges within a cylinder of radius l_{c} around it. Note that although the kernel is discontinuous, potentials are smooth thanks to the convolution with the charge density. Eventually, the material is effectively isolated: we are left with a 1D periodic system, repeated in the two additional dimensions of space in order to build potentials that mathematically fulfill 3D PBC. These modifications to the Kohn-Sham potential are then propagated consistently throughout the code in terms of total energy, forces and stress tensor and, most importantly, linear-response properties. Note that the cutoff l_{c} needs to be at least as large as the maximum distance between electrons belonging to the system, i.e., the effective thickness of the material $2t$, otherwise some physical interactions of the 1D system itself are erroneously cut. In turn, the size of the simulation cell in the non-periodic directions d should be such that electrons belonging to different periodic images are separated by at least l_{c}. In practice, the cutoff is chosen to be $l_{\mathrm{c}} = d/2$, and the supercell built such that $d > 4t$.

Computational details and model parametrization: The analytical and first-principles developments of this work are applied to the following systems: BN atomic-chain,

armchair BN nanotubes of increasing radius (i.e., (4,4,),(5,5),(6,6)), and a small wurtzite GaAs nanowire passivated with H atoms to saturate the dangling bonds on the surface (24 atoms in total). The computational details for all the calculations are those reported in the main paper. Here, we report fully in Supplementary Figure 3 the phonon dispersion relations for BN-nanotubes (5,5) and (6,6), which are used for comparison to the (4,4) tube in Figure 2 of the main text. Also, in Supplementary Table 1 we summarize the key parameter of the model (i.e., the radius) and the LO and the transverse optical (TO) frequencies at Γ to support the discussion on mechanical effects in the paper.

Referring to Figure 1 from the main text, it is worth mentioning that the dispersion of the acoustic phonons in the proximity of Γ are slightly negative and exhibit a characteristic 'wing'. These wings stem from violation of the invariance and equilibrium conditions on the lattice potential. In fact, DFPT derived interatomic force constants satisfy the proper conditions (depending on the dimensionality of the system) only approximately due to numerical convergence issues such as insufficient k-sampling or incomplete basis sets. The customary approach is then to enforce them, as a post-processing, via a specific set of relations called acoustic sum rules [10–12]. During the elaboration of this work, the suitable acoustic sum rules, restoring the correct quadratic dispersion for flexural phonons in 1D, has been implemented as described in Ref. [13].

The analytical results rely on the *ab-initio* parameters obtained independently via DFPT in 1D open-boundary conditions. The implementation of Eq. 15 requires several physical quantities. Masses, eigenvectors, eigenvalues and BECs are directly obtained from the underlying DFT and DFPT calculations. The only exceptions are the effective radius t and the dielectric tensor $\boldsymbol{\epsilon}^{\mathrm{m}}$ which is trivially replaced by its z-component (i.e., $\boldsymbol{\epsilon}^{\mathrm{m}} \rightarrow \epsilon_z \mathbb{I}$), now termed $\epsilon_{1\mathrm{D}}$. This latter choice is consistent with the isotropic assumption in our model. As discussed in the literature [14–23], the dielectric tensor concept is ill-defined in nanostructures. More advanced strategies have been proposed to model the response of two-dimensional materials [20–23] based instead on the polarizability. A particularly fundamental and robust theory has recently been proposed in 2D [24]. In this work, however, modelling the material as a dielectric cylinder implies the presence of a 1D dielectric tensor in our model. It differs from the one computed in QE, $\boldsymbol{\epsilon}^{\mathrm{QE}}$, which strongly depends on the size of the simulation cell, and the two are related via effective medium theory [14, 25] as follows. It is clear that the relevant physical quantity is the polarizability (longitudinal and transversal) which charac-

terizes the response of the isolated 1D system per unit of length. This quantity should be preserved in an isolated 1D model and in calculation with periodic images:

$$A_{\mathrm{QE}}(\epsilon_z^{\mathrm{QE}} - 1) = A_{\mathrm{1D}}(\epsilon_{\mathrm{1D}} - 1)\,, \tag{22}$$

where A_{QE} and A_{1D} stand for the cross-sectional area in the unit cell used by QE and in the 1D cylinder model, respectively. In this case, the cutoff is expected to take care of the depolarization fields coming from the periodic images [18]. The well defined physical quantity is the product $A_{\mathrm{1D}}\epsilon_{\mathrm{1D}}$. Taken individually, A_{1D} and ϵ_{1D} are just parameters of the model. There is thus a level of arbitrariness in choosing A_{1D} and ϵ_{1D} individually, keeping the product constant. Being mostly interested the long-wavelength limit $q_z \to 0$, we use the $A_{\mathrm{1D}} \to 0$ limit of this equation, while $A_{\mathrm{1D}}\epsilon_{\mathrm{1D}}$ is constant and determined by first-principles calculations. We then get:

$$\epsilon_{\mathrm{1D}} = \frac{c^2}{\pi t^2}(\epsilon_z^{\mathrm{QE}} - 1)\,, \tag{23}$$

where c is the out-of-chain length characterizing the supercell geometry (assumed to be the same in the x and y directions). Within our model, t characterizes both the electronic charge density and the out-of-chain polarization density profile. Thus, a reasonable choice to determine this parameter would be to rely on the radial electronic charge density profile, averaged on the cross-sectional planes along the 1D-axis, and set a meaningful threshold, as shown in Supplementary Figure 4. The methods for determining the radius in nanowires, nanotubes, and chains vary due to their distinct properties. In the case of nanowires and nanotubes, the maximum charge density is relatively similar, and therefore, a threshold approach is used. A threshold of approximately $\rho_{\mathrm{thr}}/\rho_{\mathrm{max}} = 10^{-3}$ is applied to obtain the radius. For chains, the charge density is typically much smaller, and the distribution profile is often similar to a Gaussian curve. In such cases, a Gaussian curve is fitted to the charge density distribution, and the radius is estimated as 2σ, where σ represents the standard deviation of the Gaussian curve. This estimation assumes that the charge density distribution is symmetrical and centered around the chain axis. In all cases, it is important to ensure that small modifications in the threshold value do not affect the quality of the results. An improved agreement of our model with DFPT could probably be obtained, in general, by replacing the isotropic approximation with the full dielectric tensor (i.e., $\epsilon_z^{\mathrm{m}} \neq \epsilon_\perp^{\mathrm{m}}$). Then, for nanotubes in particular, one could modify the choice made for the shape of the radial polarization density profile $f(\mathbf{r}_\perp)$ in Eq. 1. In fact, we are here proposing

a filled-cylinder model, while nanotubes are more likely hollow-cylinders. The reason why, despite this approximation, the model works so well may be linked to the p_z orbitals pointing inwards and thus filling the cavity of the tube. The smaller the nanotube, the more it should resemble, in this sense, a filled cylinder for our purposes. Nonetheless, since we are interested in the long-wavelength description of polar optical phonons, the agreement we get in the first tenth of the Brillouin zone is more than satisfactory even for the larger materials, and a further improvement is beyond our scopes.

II. SUPPLEMENTARY DISCUSSION

Simplified model:

Our proposed electrostatic model, presented in the main paper and in Supplementary Methods, accurately captures the 1D dielectric shift, as shown by comparison with first-principles results. Its conceptual simplicity and dependence on the radius of the 1D system, t, make it useful for spectroscopic characterization of nanostructures. However, the ill-defined nature of the macroscopic dielectric tensor in low-dimensional systems poses a challenge. To further validate our analytical results and the physical insights they provide, we propose a simpler model based on the independent-particle polarizability. This model requires less complex mathematical development and does not necessitate the definition of a microscopic dielectric tensor. However, it does involve significant simplifications in how we address the out-of-chain profile of the charge density. Let us start from the Coulomb kernel. Based on the definition of $K_0(x)$, if we Fourier-transform only along the in-chain direction, we get:

$$v_{\rm c}(\mathbf{r}_\perp, q_z) = \int_{-\infty}^{\infty} \frac{e^{-iq_z z}}{\sqrt{r_\perp^2 + z^2}}\, dz = 2K_0(r_\perp q_z)\,. \tag{24}$$

The charge density response induced by the perturbation can be rewritten as follows in terms of the independent-particle polarizability, the total potential and an out-of-chain density profile:

$$\delta\rho(\mathbf{r}_\perp, q_z) = \chi^0(q_z) V_{\rm tot}(q_z) \frac{\theta(t - |\mathbf{r}_\perp|)}{\pi t^2}\,. \tag{25}$$

In this context, we have chosen the out-of-chain profile to be a Heaviside step-function, which aligns with the main model. Thus, the induced potential is given by the convolution of the kernel and the charge density:

$$V_{\rm ind}(q_z) = \int v_{\rm c}(\mathbf{r}_\perp, q_z)\delta\rho(\mathbf{r}_\perp, q_z)\, d\mathbf{r}_\perp = \frac{4}{t^2 q_z^2} \chi^0(q_z) V_{\rm tot}(q_z)[1 - q_z t K_1(q_z t)]\,. \tag{26}$$

where, despite the finite radius charge distribution, we focus solely on what happens along the wire, specifically at $r_\perp = 0$. This further simplification arises due to the challenges posed by the Bessel functions in the kernel definition. At this point, we are interested in obtaining the dielectric function $\epsilon(q_z)$, which will be needed to get the Coulomb screened interaction entering the expression for the long-range contribution to the dynamical matrix. To get there, we can exploit the two following equations:

$$V_{\text{tot}} = \begin{cases} V_{\text{ind}} + V_{\text{pert}} \\ \frac{V_{\text{pert}}}{\epsilon} \end{cases} . \tag{27}$$

By substituting what we found so far, we get:

$$V_{\text{tot}} = \frac{V_{\text{pert}}}{\left(1 - \frac{4}{t^2 q_z^2}\chi^0(q_z)[1 - q_z t K_1(q_z t)]\right)} , \tag{28}$$

from which

$$\epsilon(q_z) = 1 - \frac{4}{t^2 q_z^2}\chi^0(q_z)[1 - q_z t K_1(q_z t)] . \tag{29}$$

Knowing that we want $v_{\text{c}}(q_z)$ to satisfy $V(q_z)_{\text{ind}} = v_{\text{c}}(q_z)\delta\rho(q_z)$, we have

$$\delta\rho(\mathbf{r}_\perp = 0, q_z) = \chi^0(q_z) V_{\text{tot}}(q_z) ,$$

leading to

$$V_{\text{ind}}(\mathbf{r}_\perp = 0, q_z) = \underbrace{\frac{4}{t^2 q_z^2}[1 - q_z t K_1(q_z t)]}_{v_{\text{c}}(r_\perp = 0, q_z)} \delta\rho(q_z) .$$

We can now recover the final expression for the Coulomb screened interaction as

$$W_{\text{c}}(q_z) = \frac{v_{\text{c}}(q_z)}{\epsilon(q_z)} = \frac{\frac{4\pi}{t^2 q_z^2}[1 - q_z t K_1(q_z t)]}{1 - \frac{4}{t^2 q_z^2}\chi^0(q_z)[1 - q_z t K_1(q_z t)]} .$$

If we assume $\chi_0 = -\alpha q_z^2$, we finally get:

$$W_{\text{c}}(q_z) = \frac{\frac{4}{t^2 q_z^2}[1 - q_z t K_1(q_z t)]}{1 + \frac{4\alpha}{t^2}[1 - q_z t K_1(q_z t)]} \tag{30}$$

and α is the macroscopic polarizability which from our *ab-initio* calculations can be obtained as (in QE $\epsilon_0 \to 1/4\pi$)

$$\alpha = \frac{A_{\text{QE}}}{4\pi}(\epsilon_{\text{QE}} - 1) .$$

The Taylor asymptotic limit for W_c of the simple model is (at leading order for $q_z \to 0$):

$$W_{c\,1\mathrm{D}}(q_z, t) = 1 - 2\gamma + 2\log(2) - 2\log(q_z t)\,. \tag{31}$$

We notice that the prefactor of the logarithm is indeed the same for the full analytical model and the simplified one. In this latter case this corresponds to $\epsilon(q_z) = 1$. The $\log(q_z t)$ term is responsible for what happens in terms of the phonon frequencies for q_z smaller than 10%, as shown in Supplementary Figure 5. For larger phonon momenta, screening becomes increasingly important and the subtleties of the full model are crucial to capture the physical behavior. In particular, we show that the full model is more successful in reproducing the DFPT predictions, despite the need of modeling the dielectric response of the system by means of $\epsilon_{1\mathrm{D}}$. We conclude that the full model offers a considerable advantage with respect to the simplified model: the mathematical complexity is slightly increased but the agreement between analytical and first-principles results is improved. The simplified model offers, however, an additional validation of the results obtained so far, shedding some light on the 1D dielectric function $\epsilon(q_z)$. For a more and rigorous approach, one would need to develop a theory, analogous to what done by Pick and Cohen for 3D systems [12] and Stengel and Royo for the 2D case [24]. In that case the radius and dielectric tensor will not be needed anymore, delivering a parameter-free model, fully based on microscopic quantities. However this goes beyond the scope of this work. In fact here one of the main goal was to provide a one-to-one relationship between frequency and size of the material, and in this case the model we propose appears to be the most rigorous and useful model we can get so far.

Experimental relevance: In the main paper we explain how the analytical model can be turned into a tool to complement and interpret photon scattering experiments on a variety of 1D systems. Here, it is instructive to follow the hLO evolution with size for a toy-model in Supplementary Figure 6: the BN atomic chain, modified by addition of atomic shells around its axis. Note that the role of t is symmetric with respect to that of q_z, meaning that the presented plot is indeed similar to the standard phonon dispersion with the difference that, instead of the phonon momentum q_z across the Brillouin zone, on the x-axis we have a possible range of radii. Then, the color-bar corresponds to the experimental laser wavelength λ which is related to the probed phonons by momentum conservation law (i.e., $q_z = 2\pi/\lambda$) . The larger the λ, the closer the probed momentum is to Γ. By increasing t,

the logarithmic overbending gradually stiffens and tends to align parallel to the energy-axis, while the short-wavelength part of the 'branch' becomes progressively flatter and wider, asymptotically converging to the 3D limit given by $\Delta\omega_{\mathrm{max}}$. These considerations are fully in agreement with what found for nanotubes in terms of DFPT. Similar considerations and possible usage of the proposed model hold as well for Raman/infrared on single, isolated, and semiconducting wires/tubes.

As a general comment, the model has been shown to perform remarkably well for different 1D prototypes (chains/tubes/wires) and different sizes. In this sense, the only caveat, if one wants to use it as a complementary tool for Raman and infrared experiments, is the accuracy of the parametrization: radius, BECs, dielectric tensor, eigenvectors and reference frequency ω_0. Depending on the size of the system, two possible strategies are viable. When dealing with small radii (e.g., the BN chain above), whose dielectric and mechanical properties are expected to differ significantly from the ones of the bulk parent, the parametrization is more delicate and computationally expensive. In fact, DFPT gives access to the above parameters at the cost of a single Γ calculation except for the q-dependency of the eigenvectors. These eigenvectors, away from Γ, lose more or less rapidly their LO character depending on the size. Consequently, additional DFPT calculations for all the required q-points involved in the spectrum are, in principle, necessary. In practice, this is crucial for smaller systems. On the other hand, for larger systems, e.g., the nanowires from Ref. [26], a different approach can be more convenient. This basically consists in assuming that the mechanical and dielectric properties required by the model are the same with respect to the bulk 3D or 2D parent, and can be extracted from a single phonon calculation at the zone center. In this case, as commented in the main text, the mechanical size-effects are only ruled by the symmetries, and the dielectric effects are fully conveyed by the radius explicitly appearing in the long-wavelength modulation $\Delta_{\mathrm{1D}}(q_z, t)$.

As far as the comparison with Ref. [26] is concerned, we further comment on the discrepancies between our predictions and experiments. Our model provides a very good agreement with the data for the larger nanowire (i.e., the base of the nanowires). Temperature effects, as well as the mixture of wurtzite and zincblende phases in the experiment, could help explaining the few cm^{-1} in excess. In fact, the LO (TO) Raman peak at 12 K is reported in the literature to be $293 \pm 1\,\mathrm{cm}^{-1}$ ($271 \pm 1\,\mathrm{cm}^{-1}$) [27] which is in agreement with our predictions at 0 K, while at 296 K is $285 \pm 7\,\mathrm{cm}^{-1}$ ($267 \pm 3\,\mathrm{cm}^{-1}$)[28], closer to Ref. [26].

Also, the branch is extremely steep in this case and the steepness seems to match well with the experimental value reported, that is the experimental data lies on the line of the model even if for slightly different q_z (see Figure 3 in the main paper). As regards the smaller nanowire (i.e., the tip), the difficulty to focus exactly the laser spot seems to be the most evident explanation behind the discrepancy: the signal from the tip and the base are not fully decoupled and experiments end up probing a mixture of signal between the two sizes. In this sense, to assess the quality of our predictions for a wide range of radii (i.e., various slopes of the LO branch) we would need new experiments on single 1D systems, isolated and constant in size. In fact, a constant radius across the material would avoid this dependency of the measured frequencies on the focus of the laser. In addition, isolated materials are needed to avoid cross-talks between the systems in the array. This happens if the spot size of the laser is comparable to the inter-systems distance, and/or for every phonon q_z smaller than the inverse of this distance. Besides these main requirements, a good control on the laser wavelength and the overall experimental set-up is needed. In fact, the non perfectly mono-chromatic nature of the laser (dependent on the type of source used) affects the set of q_z phonons effectively excited and thus contributing to the final spectrum. Similarly, depending on the penetration-depth of the exciting radiation, low-dimensional materials are known to experience a relaxation of the Raman selection rules, thus contributing to additional broadening of the spectrum. Temperature and laser heating are also crucial parameters affecting the final spectra. In conclusion, to fully validate the model and use it as simple tool to interpret experiments, a stronger interaction between the experimental and theoretical communities is desirable.

III. SUPPLEMENTARY FIGURES

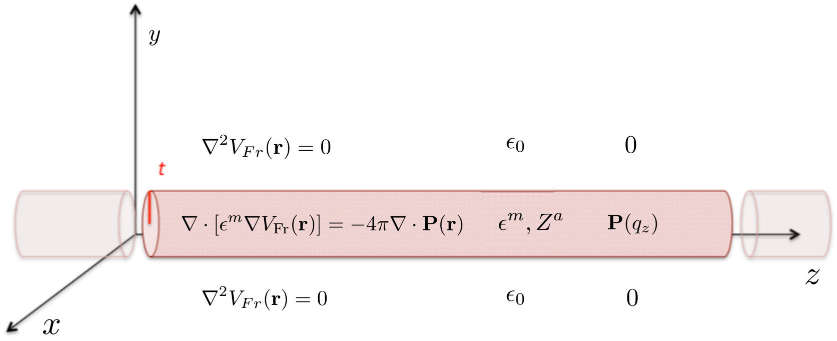

Supplementary Figure 1: Schematic representation of a 1D polar material within our model, i.e., filled cylinder of radius t. The dielectric properties ($\boldsymbol{\epsilon}^m$, $\mathbf{Z}_a$) are summarized both inside and outside the cylinder. The equations to be solved in each region are reported.

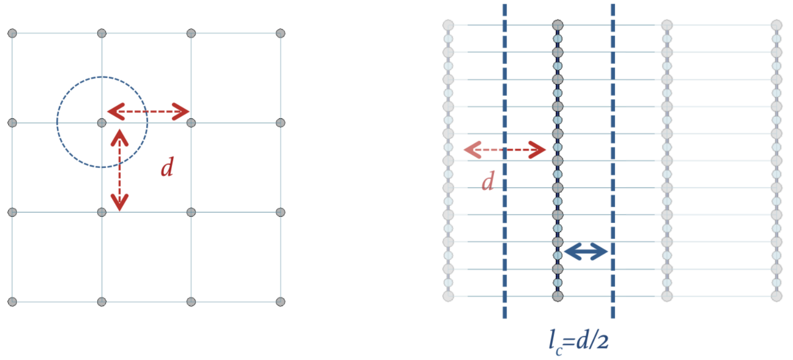

Supplementary Figure 2: Sketch of the supercell construction for 1D systems and the effect of introducing the 1D Coulomb cutoff.

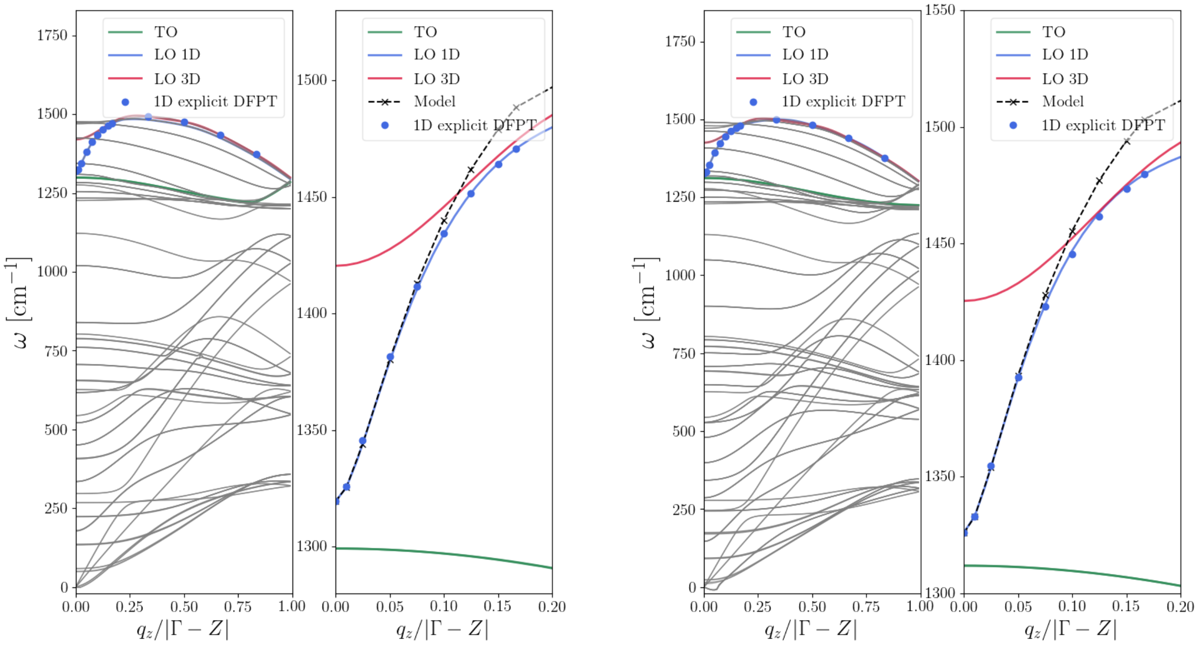

Supplementary Figure 3: Phonon dispersion relations of (5,5) and (6,6) BN nanotubes, respectively. On the left panels, we compare the *ab-initio* results using DFPT in 3D (no cutoff between periodic copies and 3D dipole-dipole long-range interactions) and using 1D-DFPT (1D Coulomb cutoff and 1D long-range interactions). On the right panels, the interpolated and explicit DFPT results are shown for long-wavelength polar-optical phonons and compared with the analytical model.

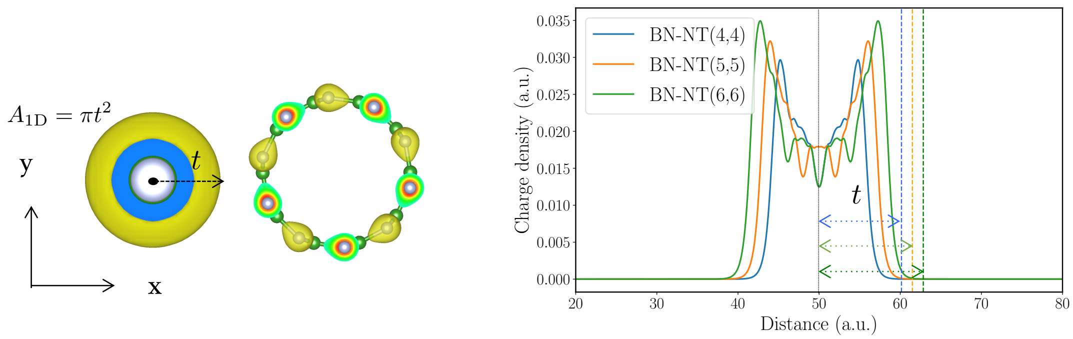

(a) (b)

Supplementary Figure 4: In the left panel (a) the radial charge density of, respectively, the BN atomic chain and one BN nanotube are shown. In the right panel (b) we report a schematic representation of how to extract the radius t from the charge density profile. The charge densities shown are obtained by averaging along the chain axis, and plotting in the radial direction. This is done for BN nanotubes of different sizes. A vertical dotted line, for each size, corresponds to the applied threshold.

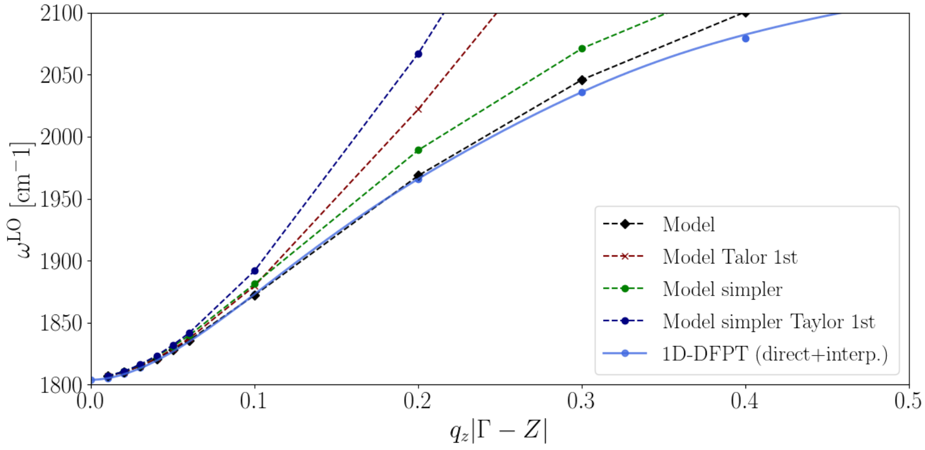

Supplementary Figure 5: Long-wavelength limit for the LO branch of the BN atomic chain. Comparison between 1D-DFPT frequencies (direct and interpolated) and the full analytical model (black) and its Taylor expansion up to the second order in $q_z t$ (red). Also the simpler model, introduced in Supplementary Discussion, is shown (green), along with its Taylor expansion (navy).

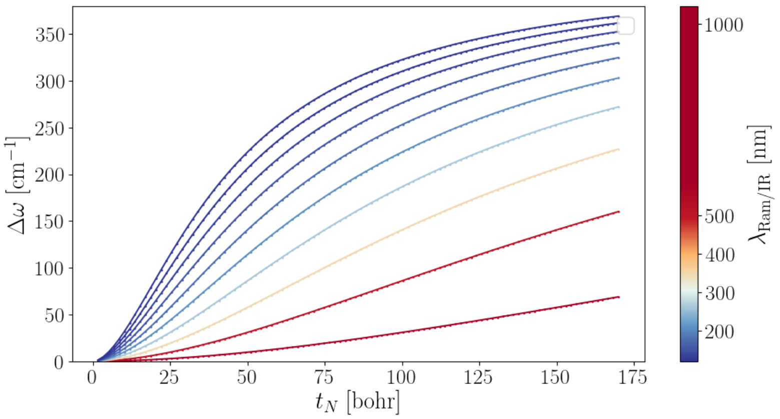

Supplementary Figure 6: Evolution of the LO branch as a function of the radius of the BN chain (toy model) obtained by the model. The color bar indicates different Raman/infrared wavelengths in experiments.

IV. SUPPLEMENTARY TABLES

1D system	t (bohr)	ω_{LO} (cm^{-1})	ω_{TO} (cm^{-1})	$\Delta\omega_{\mathrm{mech}}$ (cm^{-1})
BN-chain	1.70	1804	489	1315
BN-NNT (4,4)	10.15	1306	1275	31
BN-NNT (5,5)	11.49	1319	1299	20
BN-NNT (6,6)	12.84	1326	1312	14
GaAs-NW 24	10.41	244	240	4

Supplementary Table 1: *Ab-initio* zone-center values for ω_{LO}, ω_{TO}, the mechanical splitting $\Delta\omega_{\mathrm{mech}}$, and the effective radius t. The latter is computed by averaging the electronic density profile in the out-of-wire directions and performing a gaussian fitting for the chain (t=2σ), while setting a meaningful threshold for the other systems.

V. SUPPLEMENTARY REFERENCES

[1] Sohier, T., Gibertini, M., Calandra, M., Mauri, F. & Marzari, N. Breakdown of optical phonons' splitting in two-dimensional materials. *Nano Lett.* **17**, 3758–3763 (2017).

[2] Ismail-Beigi, S. Truncation of periodic image interactions for confined systems. *Phys. Rev. B* **73**, 233103 (2006).

[3] Rozzi, C. A., Varsano, D., Marini, A., Gross, E. K. & Rubio, A. Exact Coulomb cutoff technique for supercell calculations. *Phys. Rev. B* **73**, 205119 (2006).

[4] Castro, A., Räsänen, E. & Rozzi, C. A. Exact Coulomb cutoff technique for supercell calculations in two dimensions. *Phys. Rev. B* **80**, 033102 (2009).

[5] Sohier, T., Calandra, M. & Mauri, F. Density functional perturbation theory for gated two-dimensional heterostructures: Theoretical developments and application to flexural phonons in graphene. *Physical Review B* **96**, 075448 (2017).

[6] Baroni, S., De Gironcoli, S., Dal Corso, A. & Giannozzi, P. Phonons and related crystal properties from density-functional perturbation theory. *Rev. Mod. Phys.* **73**, 515 (2001).

[7] Giannozzi, P. *et al.* QUANTUM ESPRESSO: a modular and open-source software project for quantum simulations of materials. *J. Condens. Matter Phys.* **21**, 395502 (2009).

[8] Giannozzi, P. *et al.* Advanced capabilities for materials modelling with quantum ESPRESSO. *J. Condens. Matter Phys.* **29**, 465901 (2017).

[9] Rivano, N., Marzari, N. & Sohier, T. Density-functional perturbation theory for one-dimensional systems: implementation and relevance for phonons and electron-phonon interactions. *In preparation* (2023).

[10] Born, M. & Huang, K. *Dynamical theory of crystal lattices* (Oxford University Press, 1954).

[11] Begbie, G. H. & Born, M. Thermal scattering of X-rays by crystals-I. Dynamical foundation. *Proc. Math. Phys. Eng.* **188**, 179–188 (1947).

[12] Pick, R. M., Cohen, M. H. & Martin, R. M. Microscopic theory of force constants in the adiabatic approximation. *Phys. Rev. B* **1**, 910 (1970).

[13] Lin, C., Poncé, S. & Marzari, N. General invariance and equilibrium conditions for lattice dynamics in 1D, 2D, and 3D materials. *npj Comput. Mater.* **8**, 236 (2022).

[14] Sohier, T., Calandra, M. & Mauri, F. Two-dimensional Fröhlich interaction in transition-metal dichalcogenide monolayers: Theoretical modeling and first-principles calculations. *Phys. Rev.*

B **94**, 085415 (2016).

[15] Tóbik, J. & Dal Corso, A. Electric fields with ultrasoft pseudo-potentials: Applications to benzene and anthracene. *Chem. Phys.* **120**, 9934–9941 (2004).

[16] Yu, E. K., Stewart, D. A. & Tiwari, S. Ab initio study of polarizability and induced charge densities in multilayer graphene films. *Phys. Rev. B* **77**, 195406 (2008).

[17] Libbi, F., Bonini, N. & Marzari, N. Thermomechanical properties of honeycomb lattices from internal-coordinates potentials: the case of graphene and hexagonal boron nitride. *2D Mater.* **8**, 015026 (2020).

[18] Kozinsky, B. & Marzari, N. Static dielectric properties of carbon nanotubes from first principles. *Phys. Rev. Lett.* **96**, 166801 (2006).

[19] Tian, T. *et al.* Electronic polarizability as the fundamental variable in the dielectric properties of two-dimensional materials. *Nano Lett.* **20**, 841–851 (2019).

[20] Andersen, K., Latini, S. & Thygesen, K. S. Dielectric genome of van der waals heterostructures. *Nano Lett.* **15**, 4616–4621 (2015).

[21] Mohn, M. J., Hambach, R., Wachsmuth, P., Giorgetti, C. & Kaiser, U. Dielectric properties of graphene/MoS_2 heterostructures from ab initio calculations and electron energy-loss experiments. *Phys. Rev. B* **97**, 235410 (2018).

[22] Sohier, T., Gibertini, M. & Verstraete, M. J. Remote free-carrier screening to boost the mobility of Fröhlich-limited two-dimensional semiconductors. *Phys. Rev. Mater.* **5**, 024004 (2021).

[23] Sponza, L. & Ducastelle, F. Proper ab-initio dielectric function of 2D materials and their polarizable thickness. *arXiv preprint arXiv:2011.07811* (2020).

[24] Royo, M. & Stengel, M. Exact long-range dielectric screening and interatomic force constants in quasi-two-dimensional crystals. *Phys. Rev. X* **11**, 041027 (2021).

[25] Freysoldt, C., Eggert, P., Rinke, P., Schindlmayr, A. & Scheffler, M. Screening in two dimensions: GW calculations for surfaces and thin films using the repeated-slab approach. *Phys. Rev. B* **77**, 235428 (2008).

[26] Kim, W. *et al.* Bistability of Contact Angle and Its Role in Achieving Quantum-Thin Self-Assisted GaAs nanowires. *Nano Lett.* **18**, 49–57 (2018).

[27] Strauch, D. & Dorner, B. Phonon dispersion in GaAs. *J. Condens. Matter Phys.* **2**, 1457 (1990).

[28] Waugh, J. & Dolling, G. Crystal dynamics of gallium arsenide. *Phys. Rev.* **132**, 2410 (1963).
